# Psychometric Properties of the Lactation Assessment and Comprehensive Intervention Tool (LAT)

**DOI:** 10.3390/nursrep14040300

**Published:** 2024-12-20

**Authors:** Julie Grady, Anna Blair, Kajsa Brimdyr, Karin Cadwell

**Affiliations:** 1 School of Nursing and Health Sciences, Curry College, Milton, MA 02186, USA; 2 Healthy Children Project, Inc., Harwich, MA 02645, USA

**Keywords:** lactation, nursing, reliability, assessment, breastfeeding, LAT

## Abstract

Background: Despite the short- and long-term acknowledged benefits of breastfeeding for mothers and their infants, worldwide rates trail behind international goals. Prior research confirms that breastfeeding is a nurse sensitive indicator and that problems with latching the baby and painful breastfeeding rank high among the reasons given for not continuing to breastfeed. The Lactation Assessment Tool (LAT^TM^) was previously evaluated in a study conducted in Latvia by nurse midwives. Use of the LAT to assess breastfeeding and suggesting corrective interventions were shown to decrease pain and promote healing in traumatized nipples. The inter-rater reliability for that study was by test/re-test amongst participating researcher midwives. The aim of the current study is to expand the understanding of LAT inter-rater reliability to include novice and expert assessors. Methods: A convenience sample of twenty participants, including both novices (nine nursing students) and 11 self-identified experts, assessed four videos of breastfeeding dyads using the assessment tool, the LAT. Novice participants received a 2 h training session before final tool assessment. Each video was viewed three times, with a 3 min pause between viewings. All elements of the LAT that could be visually evaluated were included, with each element appearing in at least two of the videos. Results: Acceptable internal consistency of the LAT tool was found, with Cronbach’s alpha measuring 0.799, 0.740, 0.756 and 0.735 for each video, respectively. The reliability of the novice assessors improved over the course of the four videos, from 0.484 and 0.610 to 0.714 and 0.711. All of the experts had Cronbach’s alpha numbers that were acceptable, ranging from 0.769 to 1.00. Conclusions: Results indicate that experts perform much better using the tool than trained novices. However, the subsequent use of the tool resulted in the last two video assessments having an acceptable measure for the trained novice group. The LAT is a reliable tool for trained novices and experts to assess breastfeeding positioning and latch.

## 1. Introduction

Despite public health recommendations for increasing breastfeeding duration and exclusivity, rates trail behind international goals. The 2030 targets set by the Global Breastfeeding Collective, which includes both WHO and UNICEF, are that 70% of newborns should initiate breastfeeding in the first hour, 70% should breastfeed exclusively at 6 months, 80% should continue to breastfeed at one year, and 60% at two years. Base rates (2019) are 43% early initiation, 41% exclusive breastfeeding at 6 months, 70% breastfeeding at 1 year and 45% at 2 years. A total of 820,000 children’s lives could be saved annually if breastfeeding were to be scaled up [1]. Often overlooked are the many short- and long-term benefits for women. For example, a longer duration of breastfeeding has been associated with a lower risk of developing cardiovascular disease (CVD) in all women, but particularly those who had experienced gestational diabetes (GDM) [2,3,4]. This may be because breastfeeding has a positive effect on maternal glucose metabolism and insulin sensitivity [5,6]. In addition, meta-analyses continue to confirm the relationship between longer durations of breastfeeding and exclusive breastfeeding, and increased protections against breast cancer [7,8,9]. According to Rollins and colleagues, “not breastfeeding is associated with lower intelligence and economic losses of about $302 billion annually or 0.49% of world gross national income” [10] (p. 491). Global cost estimates by Walters and colleagues using the Cost of Not Breastfeeding Tool, found that “the largest component of economic losses, however, is the cognitive losses, which are estimated to equal US$285.4 billion annually” [11].

Breastfeeding is a nurse sensitive indicator [12] and nursing intervention strategies are needed to address problems with latching the baby and painful breastfeeding, which rank high among the reasons given for not continuing to breastfeed. Problems with latching and pain must be addressed if public health goals for breastfeeding are to be met.

A plethora of high-quality research studies elucidate the problem of difficulty latching the baby to the breast and painful breastfeeding. Wagner and colleagues interviewed more than 500 postpartum women and report that the problems of difficulty with latching the infant to the breast and breastfeeding pain were both significantly related to stopping breastfeeding or starting formula use [13]. Painful breastfeeding was also reported by Caes and colleagues as the main reason for stopping breastfeeding [14]. Feenstra and colleagues used a postal survey to explore Danish mothers’ experiences of breastfeeding [15]. They found that 40% of the 1437 surveyed population had had breastfeeding problems; problems with latching the infant on to the breast (40% of mothers) and nipple soreness (38% of mothers) were the most prominent [15]. Gianni et al. found that around 70% of the 552 mothers in their survey reported breastfeeding difficulties, including difficulty latching and cracked, painful nipples [16], which are a consequence of a poor latch. Bourdillon and colleagues’ online survey of 1084 UK mothers found that 52% of them experienced latch-related nipple pain [17]. Kronborg and colleagues’ assessment of 570 mothers identified 61% as having ineffective positioning and 52% with latch issues [18]. Thompson and colleagues, in Australia, looked at first home visit breastfeeds and reported that 411 of 653 participants (62.9%) had visible nipple trauma [19].

The role of the nurse breastfeeding care provider includes assessing and evaluating feedings, providing corrective interventions, addressing potential challenges and offering practical solutions when problems occur. “Observation of breastfeeding technique may help mothers in the stage of when they are establishing breastfeeding to avoid early and later problems” [18]. Assessment is the key tool for improving latch and decreasing the pain of sore nipples. A guidance paper for Family Physicians reminds us: “Proper positioning improves latch and reduces nipple pain” [20]. Joshi and colleagues found that effective breastfeeding positioning can have an impact beyond breastfeeding concerns, as they found positioning affected rates of diarrhea and acute respiratory infections [21]. Kronborg and Vaeth found that “ineffective positioning and latch contribute to early breastfeeding problems” [18].

Correct assessment leads to appropriate assistance. “Breastfeeding education and counseling can be designed after the initial breastfeeding assessment” [22]. Mahardika highlights that one way to increase the confidence of mothers is through breastfeeding assessment and individualized counseling [22]. Ingram’s research suggests that “those who get the technique correct when their infant is small may continue to breast feed exclusively for longer as it enhances their confidence” [23]. Patel found that breastfeeding assessment was positively associated with improved breastfeeding outcomes [24].

The LAT was created in 1997 for use by nurse midwife researchers in studies of new mothers who sought help for breastfeeding pain. The reported outcome was that assessment and corrective interventions using the LAT was related to pain reduction and healing in 95% of breastfeeding mothers with painful breasts and nipples. The tool was found to be valid [25,26]. Reliability by test/re-test was assessed before the study began for the participating nurse-midwife researchers. The potential contribution to generalizable knowledge will be the assessment of the reliability of a 24/25-item non-numerically scored breastfeeding assessment tool.

Unlike other breastfeeding assessment tools which use a numeric scoring system [27,28,29,30], the LAT identifies 25 items associated with pre-feeding state of the baby, positioning at the breast, latching, suckling and ending the feeding, and allows for the assessor to choose ideal and less than ideal options for each item. After the assessment, the tool is consulted to determine potential corrective interventions. The items are grouped into five sections for ease of use: pre-feeding, during latching-on, during feeding, post-feeding, and mother’s comfort level. Mother’s comfort level cannot be assessed in the video format, so it is not included in this reliability study, this elimination results in twenty-four (24) individual items in four categories to be included in the analysis.

Pre-feeding: The first item in the pre-feeding group is whether the baby is skin-to-skin immediately prior to/during this current feeding. Breastfeeding problems are resolved more quickly when the baby is placed skin-to-skin before a breastfeeding attempt. “With skin-to-skin contact, the infant can relax, stop crying and maintain enough calm to successively, and successfully, co-ordinate body movements with the five senses (sight, touch, hearing, smell and taste) to achieve the latch and suckle, echoing the patterns of a normal newborn during the first hours of life” [31]. Pre-feeding also includes an assessment of the state of the baby. The assessor determines if the baby is in deep sleep, light sleep, quiet/active alert or crying [32,33,34,35,36]. Assessors determine readiness to feed by assessing whether there are feeding cues which could include rooting and/or hand to mouth actions [25].

The group of items involving body position and the latching process are grouped under the heading “During Latching”. Body position includes the alignment of the head, shoulders and hip [37], the position of the baby’s arms in relation to the breast [38], whether the baby’s head is free of restrictions [19], and whether the breast is held or shaped [19]. The latching process involves assessing the baby’s nose position in relation to the nipple [37,39], whether the baby gapes, the head tilt of the baby during the gape, and whether the bottom lip and tongue make contact with the breast first [40,41,42].

During the feeding, the assessment includes the position of the nose, chin and cheeks [37], the angle of the mouth [37], the seal of the top and bottom lip [37,43,44,45,46], whether the cheek is rounded [47], and whether there is a dimple [47]. The assessor also evaluated the number of sucks per swallow [43,47], whether the latch is symmetric or asymmetric [37], and whether the jaw motion is considered to be a rocker motion or a piston motion [37,40]. The assessor notes who releases the nipple, and the tone of the baby at the end of the feeding.

Although validity testing has been performed in the past with nurse-midwives, this study aims to determine inter-rater reliability for the LAT when used by novice and expert assessors.

## 2. Materials and Methods

After receiving IRB approval, content experts (AB and KB) selected four (4) videos for analysis and determined the items of the LAT that could be assessed in each video. KC and JG were blinded to the selected elements. Table 1 represents the items of the LAT that were eligible to be assessed in each video and the number of times each would be assessed in total. “Mother’s Comfort Level” (item 25) was unable to be assessed since the assessments were being done with video, as it was not possible to ask the mothers any questions.

Both novices and experts were recruited to view the videos and assess the feedings using the LAT. A convenience sample of students who elected to participate in an educational program about assessing breastfeeding constituted the novices, none of whom had worked clinically providing breastfeeding support. Experts were recruited at a conference if they elected to attend a session on positioning and latch and considered themselves experts.

A convenience sampling strategy was utilized in the hopes that that this could increase the generalizability of the results by increasing the diversity of participants. Novice participants were recruited by utilizing a preplanned recruiting script. Student nurses (undergraduate and graduate) were recruited using flyers, word of mouth, and snowball sampling. Participation in the educational offering was voluntary. Prior to beginning the educational offering, informed consent was obtained. The inclusion criteria were that nursing students (undergraduate and graduate) had completed a nursing health assessment course. Subject matter experts were invited at a breastfeeding conference to attend the breastfeeding assessment workshop based on their credentials. Inclusion criteria were that they had a professional credential in lactation and had previous experience assessing breastfeeding using assessment tools.

Nine (9) nursing students consented to participate in the education offering. The students represented a diversity of educational experience, and consisted of three (3) traditional pre-licensure who were in their junior year, five (5) accelerated pre-licensure who were in the third semester of a four-semester program, and one (1) post-licensure master’s degree student who was in the fourth semester of a six-semester program. A convenience sample of eleven (11) subject matter experts consented to participate in the LAT assessment workshop, representing a diversity of backgrounds and experience. The experts consisted of five (5) Certified Lactation Counselors (CLCs), five (5) Internationally Board-Certified Lactation Consultants (IBCLCs), and five (5) Advanced Nurse Lactation Consultants (ANLCs). Six (6) were registered nurses (RN) (one APRN), one (1) PhD, five (5) with master’s degrees, one (1) social worker, and one (1) mental health therapist. (It is possible to hold multiple credentials.) All had previously used lactation assessment tools, with two having less than 10 years clinical breastfeeding experience, three having 10–20 years clinical breastfeeding experience, and six having more than 30 years clinical breastfeeding experience. In total, twenty (20) participants, both novice and expert, were included in the study.

Novice participants were provided with a program of education by a content expert (KC) including a lecture, the use of PowerPoint presentations and videos related to the assessment of breastfeeding sessions using the LAT. Included were aspects of pre-feeding, during latch-on, during feeding and following the feeding. The students practiced their assessment skills by viewing videos. Discussion allowed the students to ask questions and clarify their understanding concerning breastfeeding and the use of the LAT. Students then assessed the four videotapes of breastfeeding sessions prepared for the study.

No review was provided to expert assessors prior to assessing the study videos.

The four selected videos were shown to the participants, in person, in a classroom setting. Each of the videos was shown separately, with a 3 min opportunity to fill out the LAT. Discussion was not permitted during the assessments by either the novices or the experts until all the LAT tools were collected by an investigator. The paper copies of the LAT tool were identified by the number of the video and whether the assessor was in the novice or expert group.

After data entry was verified, the reliability of the tool was calculated for each of the four videos using SPSS version 26.

### Data Analysis

The marked items on the collected LAT tools were entered by the primary investigator into Excel. Data entry was confirmed to minimize data entry errors by a second investigator. Novice and expert assessments were analyzed together and separately. Statistical Package for the Social Sciences (SPSS) software version 26 was used to summarize descriptive variables and to analyze each of the four (4) videos. Cronbach’s alpha was used to analyze the inter-rater reliability of the LAT instrument. Cronbach’s alpha is a widely used method of internal consistency and reliability [48].

The electronic data collected are stored on the external password-protected hard drive in the primary investigator’s home office. The data are only accessible by the primary investigator and are continuously password protected. All data will be destroyed within the five years following the submission of the findings.

Institutional Review Board (IRB) approval was obtained from Curry College, the academic institution where the educational offering for novices was conducted. The LAT forms were anonymous and labeled with numbers for analysis purposes only and were not identifiable to the participant.

Consent was obtained prior to beginning the in-person educational offering for novices and prior to viewing and assessing the videos for the experts. The educational offering was voluntary and the participants were informed that they could choose not to participate and/or to leave at any time without completing the education and/or the final review of the videos including the use of the LAT. There was no foreseeable risk associated with participation in this study and strict anonymity was maintained following the collection of the data. As this was an in-person educational offering for the students, those students who participated were aware of other students’ participation. The participation of experts was also in-person, and participants were aware of others’ participation.

## 3. Results

The LAT assessment results of both novices and experts were analyzed separately and in combination for each video. The Cronbach’s alpha scores ranged from 0.735 to 0.799. Table 2 displays the scores associated with each of the videos, as well as the Cronbach’s alpha scores of the novice and experts.

## 4. Discussion

A recent Cochrane Review concludes that breastfeeding support is likely to increase the duration and exclusivity of breastfeeding [49]. Many of the studies included in the review point to practical support, assessing and providing help with positioning and latch, as the key components of “support”. It is not surprising, then, that multiple tools have been developed and evaluated for reliability. Each tool defines and seeks to assess breastfeeding in different ways. All but the LAT include multiple aspects in each sub-score and assign a total numerical score to the assessment.

The Bristol Breastfeeding Assessment Tool (BBAT) includes four items: positioning, attachment, sucking and swallowing. The inter-rater reliability was conducted with seven midwives, and the subsequent assessment on 160 dyads found inter-rater reliability Cronbach’s alpha to be 0.782 [29].

Riordan and Koehn conducted inter-rater reliability testing for the Infant Breastfeeding Assessment Tool (IBFAT), the LATCH, and the Mother Baby Assessment Tool (MBA). The test participants were three nurse raters, using 23 video assessments of breastfeeding dyads. The study found that “These results indicate that the IBFAT, the LATCH assessment tool, and the MBA are not sufficiently reliable in their current form for clinical practice.” [50] The percentage of agreement among raters for each variable was ranged from 37.0 to 97.2. More recently, researchers have found positive and significant correlations between the three tools [51].

The IBFAT includes five items, assessing whether the baby is wakeful and ready to feed, showing feeding cues (rooting), the length of time from latch to sucking, and the sucking pattern. The tool asks raters to choose a score between 0, 1, 2, and 3 for each item. The tool also includes asking the mother how she felt about the feeding [28]. Inter-rater reliability between the three assessors varied from 62% for the question of whether the baby was ready to feed/needed stimulation, to 87.5% for the length of time from latch to suckling. Riordan and Koehn note that 80% is the cut-off for an acceptable minimum agreement. Only two of the IBFAT 5 elements had above 80% agreement by the three nurse assessors; length of time from latch to suckling and presence of feeding cues/rooting. The other three elements fell below 80% agreement [50].

Modeled after the Apgar score, the LATCH tool has been used in research and practice, examining latch, swallowing, the type of nipple, comfort and hold [27]. The tool asks raters to choose between 0, 1, and 2 for each item. As assessed by Riordan and Koehn, the inter-rater reliability between the three nurse assessors ranged from 54.2% to 97.2%. Only “assistance needed in holding the infant” fell below the 80% cut-off, with 54.2% agreement [50]. Recently, the LATCH tool has been used to analyze the impact of breastfeeding supportive measures for dyads in the early postpartum period. In total, 99.7% (399) of 400 mother–infant dyads in the study required support to position the neonate, 47.5% (190) had a poor latch and 13% (52) were found to have nipple problems during the assessment. Breastfeeding support measures were found to improve the participant’s LATCH scores [52].

The MBA tool assesses readiness to feed, positioning, latch, milk transfer, and outcome [30]. The assessors choose between 0 and 1 for the mother and for the infant for each of the five elements. As assessed by Riordan and Koehn, the inter-rater reliability between the three nurse assessors ranged from 37% to 95.2%. Three elements were assessed above the 80% cut-off: readiness to feed, positioning and latch.

The BBAT does not include pre-feeding behaviors. The IBFAT does not include positioning of the baby or the latching process. LATCH is primarily a hospital-based tool, and does not include pre-feeding or the latching process. The MBA tool does not assess the latching process. None of these tools assess the state of the baby before or after the feeding, or the state of the breast after the feeding. All of these tools group multiple aspects of a breastfeeding session together into one sub-score and a final total score.

The 24 elements of the LAT provide the assessor with the opportunity to analyze each of the discrete elements of the breastfeeding session; beginning with pre-feeding, during the latching process, during feeding and post-feeding including the condition of the breast, the state of the baby at the end of the feeding and the mother’s comfort level. The only element not assessed in this study was “Mother’s Comfort Level”. However, previous studies utilizing the LAT have demonstrated a decrease of the mother’s pain when the LAT is used to correct and improve the breastfeeding experience [26].

The study presented here has some limitations, such as potential selection bias, sample size of the novices and experts and lack of heterogeneity. As a convenience sample from one academic setting for the nursing student novices, and one conference for the self-identified experts, results may not be generalizable to other settings. In addition, there were a limited number of breastfeeding dyads assessed, although the improvement on the part of the novices towards a similar Cronbach alpha to the experts lends itself to confidence that the number of videos was sufficient. The breastfeeding dyads assessed were on video, instead of in-person assessing/counseling, which could be seen as a limitation but could also be a strength, given the use of telehealth in today’s health care system. An advantage of the pre-recorded videos was that all assessors could observe the same view during the assessment, and that the video could be repeated multiple times and in multiple settings. A limitation of a pre-video is that it is not possible to ask the mother questions, such as her pain level. However, the LAT had previously be used in a study about positioning and pain and there was a relationship between improvements in the LAT and the improvements in pain levels. For future research, it is important to be aware of the limitations of this study.

Even though the LAT has significantly more items for assessment, after a 2 h training session, novice participants demonstrated acceptable inter-rater relatability for the LAT illustrating its ease of use, even among novice nursing students. The LAT can be used for early, middle and later breastfeeding assessments, not only during a hospital stay.

## 5. Conclusions

The study tool, the LAT, was previously evaluated for inter-rater reliability as part of a research project using the test/re-test method of the nurse midwife researchers in the study, and subsequently used with 92 of the breastfeeding dyads who participated in the study. Inter-rater reliability was conducted, but not published [25]. The current inter-rater reliability of the LAT, conducted with both novice and expert breastfeeding assessors, includes significantly more assessors than other breastfeeding assessment tools. The Cronbach’s alpha scores of the four breastfeeding videos for assessment are all above 0.7 (0.799, 0.740, 0.756 and 0.735), which shows acceptable internal consistency. The LAT can and should continue to be used clinically to guide nurses and other breastfeeding care providers as they assess and provide corrective interventions for the breastfeeding dyad in order to improve breastfeeding duration and concomitant health outcomes.

## Figures and Tables

**Table 1:** Summary of which items in the LAT are assessed in each video.

Item	Number of Times Assessed	Video 1	Video 2	Video 3	Video 4
Pre-feeding					
Skin-to-skin	4	X	X	X	X
State of baby	4	X	X	X	X
Feeding cues exhibited	2	X	X		
During latch-on					
Body position					
Tummy to mummy	3		X	X	X
Baby arms/hands around breast	2			X	X
Baby’s head free of restrictions	2	X		X	
Breast is not held or shaped	2	X			X
Latching process					
Nose opposite nipple	4		X	X	X
Gape response	3		X		X
Head tilts back during gape	4	X	X	X	X
Bottom lip and tongue reach breast first	3		X	X	X
During feeding					
Facial proximity					
Nose, chin and both cheeks close to breast	2	X			X
Angle of mouth opening					
Greater than or equal to 140 degrees +	3		X	X	X
Mouth seal					
Top and bottom lip sealed	4		X	X	X
Rounded cheek line	3	X	X		X
Not dimpled cheek	3	X	X		X
Rhythm					
Bursts of 2:1 or 1:1	3		X	X	X
Latch type					
Asymmetric	3		X		X
Jaw motion					
Rocker	2		X	X	
Post-feeding					
Ending feeding					
Baby releases nipple	4	X	X	X	X
Baby’s tone					
Soft tone and/or satiated	3	X	X	X	
Relaxed hands	2		X		X
Mother’s nipple					
Similar to pre-feed/undamaged	2	X			X
Mother’s comfort level					
Tugging feeling—not able to assess With videos					

**Table 2:** Reliability scores: Cronbach’s alpha of the LAT for each of the assessed videos.

	Video 1	Video 2	Video 3	Video 4
Combined novice and expert	0.799	0.740	0.756	0.735
Novice	0.484	0.610	0.714	0.711
Expert	0.916	0.946	1.00	0.769

## Data Availability

Data are available upon reasonable request from the corresponding author.

## Public Involvement Statement

No public involvement in any aspect of this research.

## Guidelines and Standards Statement

This manuscript was drafted against the Recommendations for reporting the results of studies of instrument and scale development and testing [53] (for reliability research.
